# Structural Characterization and Hypoglycemic Activity of a Glycoprotein Extracted from *Auricularia Auricula*

**DOI:** 10.3390/foods13233859

**Published:** 2024-11-29

**Authors:** Qiping Zhan, Mengdie Yang, Xinqi Zhao, Feifei Liu, Liyan Zhao

**Affiliations:** College of Food Science and Technology, Nanjing Agricultural University, Nanjing 210095, China; qipingzhan@njau.edu.cn (Q.Z.); 2020108073@njau.edu.cn (M.Y.); zhaoxinqi@stu.njau.edu.cn (X.Z.); 2023108081@stu.njau.edu.cn (F.L.)

**Keywords:** *Auricularia Auricula*, nutrient, glycoprotein, structural characterization, hypoglycemic activity

## Abstract

Glycoproteins are special proteins and important nutrients for hypoglycemic activity. However, the structure of *Auricularia Auricula* glycoprotein (AAG) and the stability of its hypoglycemic activity during simulated digestion (including saliva, gastral and intestine digestion) in vitro are still unknown. In this study, AAG-3 was isolated from *Auricularia Auricula*. SDS-PAGE, UV spectrum, FTIR, amino acid composition, dichroic spectrum and SEM were used to characterize its structure. The hypoglycemic activity of AAG-3 during in vitro digestion was investigated via inhibition of α-amylase and α-glucosidase activities, as well as glucose consumption, glycogen content and related enzyme activity in insulin-resistant HepG2 cells. Structural characterization showed that AAG-3 with a Mw of 18.21 kDa had an O-type glycopeptide bond and typical functional groups of glycoproteins. AAG-3 contained 18 kinds of amino acid and many α-helixes and β-turns, and its microstructure was sheet-like. With the simulated digestion of AAG-3 in vitro, the inhibition of α-amylase and α-glucosidase activity as well as the glucose consumption, glycogen content and HK and PK enzyme activities in insulin-resistant HepG2 cells were significantly increased. Therefore, AAG-3 has a potential role in reducing blood glucose levels and improving insulin resistance and can be used as a potential micronutritional supplement for diabetic patients.

## 1. Introduction

Type 2 diabetes mellitus (T2DM) is a chronic metabolic disease characterized by hyperglycemia and insulin resistance, with serious complications leading to high mortality [1,2,3]. T2DM has a high incidence, with nearly 90% of the 537 million cases worldwide being T2DM [2,3]. Currently, oral antidiabetic drugs can be categorized into different types based on their different targets and mechanisms of action, such as α-glucosidase inhibitors (A-Caboose, etc.), insulin sensitizers (melbine, etc.) and so on. Although these drugs have certain therapeutic effects, long-term use can lead to serious side effects [1], including hypoglycemia, osteoporosis, heart failure, potential toxicity and so on [2]. Therefore, there is an urgent need to search for more effective, safe and well-tolerated natural hypoglycemic active ingredients [2]. Some in vitro experiments such as α-amylase and α-glucosidase inhibition experiments and insulin-resistant HepG2 cells are widely used to screen active ingredients with hypoglycemic activity.

The α-amylase secreted by the salivary glands or pancreas breaks down starch into glucose, which is then released into the blood. Additionally, α-glucosidase, secreted by the small intestine, breaks down maltose or sucrose into glucose, also releasing it into the blood [3]. That causes postprandial hyperglycemia and exacerbates non-insulin-dependent or type 2 diabetes [3]. The liver is responsible for about 90% of endogenous glucose production, and increased hepatic gluconeogenesis may be the cause of hyperglycemia, which ultimately exacerbates insulin resistance [2]. Insulin-resistant HepG2 cells are a reliable model to investigate the potential hypoglycemic activity of active components. Hexokinase (HK) and pyruvate kinase (PK) are key rate-limiting enzymes in HepG2 cells, which are related to the rate of glycolysis and are important indexes for studying glucose metabolism in insulin-resistant cells [2]. Therefore, the potential hypoglycemic activity of the active component can be investigated via α-amylase and α-glucosidase inhibition assays as well as glucose consumption, glycogen content, glycogen PAS staining and HK and PK enzyme activities assays in insulin-resistant HepG2 cells [2]. These selected hypoglycemic active ingredients provide a strong guarantee for people to improve postprandial blood glucose through diet to prevent the occurrence of T2DM.

The intake of nutrients is very important for promoting healthy glucose metabolism in diabetes patients [4]. It has become a new demand of national health to prevent diabetes through dietary nutrition. Glycoproteins are a kind of special nutrient, which have good physiological functions, especially hypoglycemic activity [5]. Glycoproteins are polysaccharide–peptide or polysaccharide-protein complexes, of which protein molecules usually carry one or more carbohydrate chains through N- or O- covalent bonds [6,7]. Glycoproteins usually have the biological activity of both polysaccharides and proteins [8] and play an important role in macrophages, recognize pathogens and activate immune responses [9]. The surface glycoproteins (such as APMAP) on THP-1 monocytes and macrophages participate in responses to bacterial infection and during the monocyte-to-macrophage differentiation to enhance the immune responses of macrophages [9]. Research showed that food-derived glycoproteins have hypoglycemic, hypolipidemic, anti-inflammatory, antimicrobial, antioxidant, immunomodulatory and anticancer properties [1,8,10]. In addition, the functional stability of glycoproteins during gastrointestinal digestion is also an important aspect to study the functional activity of glycoproteins [3]. Edible fungi have good nutritional and medicinal value, rich in polysaccharide and protein, and are a good potential source of glycoproteins [11]. *Auricularia Auricula* is collected or cultivated on a large scale in many countries, including Korea, China, Russia and Japan, and is listed among the four largest edible mushroom varieties in the world [12]. *Auricularia Auricula* is an edible fungus with significant nutritional and pharmacological benefits. It is particularly rich in polysaccharides, proteins, glycoprotein, lipids, vitamins, pigments and trace elements, which contribute to its substantial nutritional value [12]. At present, studies on the active constituents of *Auricularia Auricula* mainly focus on polysaccharide, melanin, polyphenols, sterols, alkaloid and protein [12,13]. There is only one study on *Auricularia Auricula* glycoprotein (lectin) inhibiting the proliferation of lung cancer cells [14]. *Auricularia Auricula* is recognized for its biological activities such as anti-fatigue, antibacterial, anticoagulant, hypoglycemic activities and so on [12,14,15,16]. *Auricularia Auricula* has particularly good hypoglycemic activity, but the research on the effective components of its hypoglycemic activity mainly focuses on polysaccharide rather than glycoprotein [15,16]. Therefore, this study aims to systematically explore the hypoglycemic activity of *Auricularia Auricula* glycoprotein, enrich people’s cognition of the effective ingredients of hypoglycemic activity in *Auricularia Auricula* and promote the high-value utilization of *Auricularia Auricula* glycoprotein in hypoglycemic functional foods.

In this study, a glycoprotein, named *Auricularia Auricula* glycoprotein-3 (AAG-3), with a purity of >96%, was isolated from *Auricularia Auricula*. The structure of AAG-3 was characterized via sodium dodecyl sulfate-polyacrylamide gel electrophoresis (SDS-PAGE), the ultraviolet (UV) spectrum, Fourier transform infrared spectroscopy (FT-IR), amino acid composition, the circular dichroic (CD) spectrum and scanning electron microscope (SEM) analysis. The hypoglycemic activity of AAG-3 during in vitro digestion was evaluated by investigating its effects on the inhibition of α-amylase and α-glucosidase activity, as well as glucose consumption, glycogen content, glycogen PAS staining and HK and PK enzyme activity in insulin-resistant HepG-2 cell models. This study provides important implications for the use of AAG-3 as a functional food to prevent the occurrence of T2DM or improve the blood glucose level of diabetic patients and provides a theoretical basis for further exploration.

## 2. Materials and Methods

### 2.1. Chemicals and Reagents

The *A. auricula* samples were purchased from Henan Gutailang Foods Co., Ltd. (Henan, China). DEAE-Sepharose Fast Flow and Sephadex G-75 were purchased from GE Healthcare Life Sciences (Uppsala, Uppland, Sweden). Coomassie brilliant blue R-250 (≥AR) was purchased from Shanghai Yuanye Bio-Technology Co., Ltd. (Shanghai, China). Bovine Serum Albumin (BSA, 96%) was purchased from Shanghai Macklin Biochemical Co., Ltd. (Shanghai, China). NuPAGE Novex Bis-Tris 10% resolving gel and NuPAGE MES-SDS running buffer were purchased from Invitrogen (Paisley, Scotland, UK), and α-amylase was purchased from Shanghai Ruiyong Biotechnology Co., Ltd. (Shanghai, China). DMEM, fetal bovine serum (FBS) and dual antibody were purchased from Thermo Fisher Technology Co., Ltd. (Beijing, China), and 0.25% trypsin solution, HepG2 cells, CCK-8 kit, glycogen content determination kit, and HK and PK activities kits were purchased from Beijing Solaibao Technology Co., Ltd. (Beijing, China). α-glucosidase and hematoxylin staining solution were purchased from Shanghai Yuanye Biotechnology Co., Ltd. (Shanghai, China). Insulin was purchased from Shanghai MikeLin Biochem Technology Co., Ltd. (Shanghai, China).

### 2.2. Extraction of Crude A. auricula Glycoprotein (AAG)

The combination of isoelectric-ammonium sulfate precipitation was used to extract crude *A. auricula* glycoprotein (AAG) [13]. NaOH alkaline extraction (pH 10) combined with 1 M NaCl suspensions of *A. auricula* meal was prepared (meal/solvent ratio: 1:70 *w*/*v*). After extraction for 3 h, the slurries were centrifuged at 1776× *g* for 20 min at 4 °C, and then, the supernatants were separated and the solubilized glycoprotein in the supernatant was precipitated with ammonium sulfate at a saturation of 70%. The precipitate was obtained via centrifugation in the aforementioned condition, and the pellets were rinsed, collected, dispersed and dialyzed in deionized water at 4 °C for 48 h. The dialyzed liquids were freeze-dried to obtain AAG.

### 2.3. Purification of AAG

#### 2.3.1. DEAE-Sepharose Fast Flow Column Chromatography

Purification of AAG using a DEAE-Sepharose Fast Flow chromatography column was conducted referring to the method of Tsai with some modifications [17]. First, 0.25 g AAG was dissolved in 25 mL Tris-HCl buffer (pH 8.0, 20 mM) and run through a DEAE-Sepharose Fast Flow chromatography column (2.6 × 30 cm), which had been equilibrated with Tris-HCl buffer. Protein-rich fractions were collected through 0–0.7 M NaCl stage Tris-HCl buffer, the flow rate of which was 1.8 mL/min. Finally, the column was rinsed with 2 M NaCl Tris-HCl buffer. The effluent was collected using an automatic collector with 8 mL each tube. After that, the absorbance of protein was measured at 280 nm, while the absorbance of carbohydrate was measured at 490 nm via the phenol-sulfuric acid method. The elution solution of protein and carbohydrate with absorption peaks at both wavelengths was collected. Four fractions, AAG-1, AAG-2, AAG-3 and AAG-4, were obtained, dialyzed and lyophilized. In this study, we mainly focused on AAG-3.

#### 2.3.2. Sephadex G-75 Column Chromatography

Purification of AAG-3 via Sephadex G-75 column chromatography was conducted referring to the method of Zeng with some modifications [18]. First, 20 mg of AAG-3 was dissolved in 2 mL of distilled water and run through a 1.6 × 60 cm Sephadex G-75 chromatography column, which had been equilibrated with distilled water. Protein-rich fractions were collected, the flow rate of which was 0.67 mL/min. The effluent was collected using an automatic collector with 2 mL each tube. After that, the absorbance of protein was measured at 280 nm, while the absorbance of carbohydrate was measured at 490 nm using the phenol-sulfuric acid method. The elution solution of protein and carbohydrate with absorption peaks at both wavelengths was collected and lyophilized.

### 2.4. Structural Analysis

#### 2.4.1. Protein Content and Carbohydrate Content Analysis

The protein content of AAG-3 was determined following the method of Lowry with BSA as the standard [19]. The carbohydrate content of AAG-3 was determined using the phenol-sulfuric acid method with glucose as the standard [7].

#### 2.4.2. SDS-PAGE Analysis

Using the method of Ramos-Pineda with some modifications, 10% SDS-PAGE was carried out [20]. A total of 10 mg AAG-3 prepared in 1 mL distilled water under reducing condition with DTT was used for SDS-PAGE. First, 5 μL of each sample was loaded into a NuPAGE Novex Bis-Tris 10% resolving gel. The electrophoresis was carried out using NuPAGE MES-SDS, running the buffer at room temperature. After that, the gel was stained with 2.5 g/L Coomassie brilliant blue R-250 staining solution for 1 h and then de-stained in 10% acetic acid overnight. The electrophoretic gels were scanned using a white light transilluminator screen in a Gel Doc XR+ imaging system (Bio-Rad Laboratories, Hercules, CA, USA). Bands were analyzed using Image Lab software (version 5.1, Bio-Rad Laboratories, Hercules, CA, USA). The relative mobility (Rf) was calculated by measuring the migration distance (pixel) of the protein band and the migration distance (pixel) of the dye front end.

Rf = protein band migration distance/dye front migration distance

With the logarithm of the molecular weight of protein standards from 180 kDa to 10 kDa as the ordinate and the relative mobility as the abscissa, the linear fitting curve was obtained to estimate the molecular weight (Mw) of AAG-3.

#### 2.4.3. Amino Acid Composition

The amino acid composition of AAG-3 was determined according to the method of Qin [3]. AAG-3 was hydrolyzed in 6 M HCl at 110 °C for 24 h using a Hitachi Model L-8900 Amino Acid Analyzer (Hitachi Ltd., Tokyo, Japan).

#### 2.4.4. UV Spectrum

To determine the type of glycosylation, 2 mg AAG-3 was dissolved in 10 mL NaOH solution (0.2 M) and incubated at 45 °C in a water bath for 3 h, followed by UV analysis using a TU1900 UV spectrophotometer ranging from 200 cm^−1^ to 400 cm^−1^. The sample without NaOH was used as the control [7].

#### 2.4.5. FTIR Spectrum Analysis

The infrared spectra of AAG-3 (1 mg) was analyzed according to the potassium bromide disk method using a FTIR instrument (Thermo Fisher Scientific, NICOLET IR200, Waltham, MA, USA) in the frequency range of 4000–400 cm^−1^ [21].

#### 2.4.6. Circular Dichroic (CD) Spectrum Analysis

For CD spectrum analysis, 0.3 mg of AAG-3 was dissolved in 1 mL deionized water. The scanning wavelength was 190–260 nm, the bandwidth was 1 nm, and the scanning speed was 20 nm/min. Each spectrum was scanned three times. Subsequently, all spectra underwent smoothing, and background deduction was carried out with respect to deionized water. The secondary structure content were subjected to analysis using CDNN and K2D2 software (version 2.1) [22].

#### 2.4.7. SEM Analysis

The ultrastructure of AAG-3 was observed using a scanning electron microscope (SEM, Philips XL-30, Philips-FEI Co., Eindhoven, The Netherlands). The sample was coated with gold powder and placed on a specimen holder. The sample was observed with 3000-fold magnification at a voltage of 3.0 kV under high-vacuum conditions [23].

### 2.5. In Vitro Hypoglycemic Activity of AAG-3

#### 2.5.1. In Vitro Simulated Digestion of AAG-3

The simulated saliva, simulated gastric juice and simulated small intestinal juice were prepared according to the method of Ding [24]. The simulated saliva digestion, gastric digestion and intestinal digestion were carried out according to the reported method with some modifications [24]. The control group was treated the same without AAG-3. Each experiment was replicated independently three times.

##### Simulated Saliva Digestion

First, 0.05 g AAG-3 was added to 1 mL simulated saliva and stirred at 37 °C for 5 min, and the reaction was terminated by heating to 85 °C to inactivate enzymes. The reaction solution was dialyzed and lyophilized to obtain AAG-3-S for subsequent analysis.

##### Simulated Gastric Digestion

The reaction solution after the simulated saliva digestion was adjusted to 2.0 using 0.1 M HCl solution. Then, 10 mL simulated gastric juice was added and stirred at 37 °C for 60 min, and the reaction was terminated by heating to 85 °C to inactivate enzymes. The reaction solution was dialyzed and lyophilized to obtain AAG-3-G for subsequent analysis.

##### Simulated Intestinal Digestion

The reaction solution after the simulated gastric digestion was adjusted to 7.0 with 0.1 M NaOH solution. Then, 10 mL simulated gastric juice was added and stirred at 37 °C for 60 min, and the reaction was terminated by heating to 85 °C to inactivate enzymes. The reaction solution was dialyzed and lyophilized to obtain AAG-3-I for subsequent analysis.

#### 2.5.2. The Inhibitory Effect of AAG-3 During In Vitro Digestion on α-Amylase

The α-amylase inhibitory activity was performed according to the method of Qin with some modifications [3]. The samples, enzymes and substrates used in the reaction were dissolved in 0.1 mol/L PBS (pH 6.8). Then, 100 μL of α-amylase solution (40 U/mL), 100 μL of 1% starch solution and 100 μL of AAG-3/AAG-3-S/AAG-3-G/AAG-3-I (3 mg/mL) or different concentrations of AAG-3-I (4, 6, 8, 10, 16, 20 mg/mL) were incubated at 37 °C for 15 min. After incubation, 100 μL of 0.01 M iodine solution and 500 μL of deionized water were added. The absorbance was measured at 660 nm. The blank control group, control group and blank group refer to the no α-amylase and glycoprotein sample, no glycoprotein sample and no α-amylase in the system, respectively. The absorbance was measured at 660 nm, and the inhibition rate (R) of α-amylase was calculated as follows:

X = (A_00_ − A_11_)/A_00_ × 800

Y = (A_0_ − A_1_)/A_0_ × 800

R = (X − Y)/X × 100%

where X is the total enzyme activity, and Y is the enzyme activity after inhibition. A_00_, A_11_, A_0_ and A_1_ represent the absorbance values of the blank control group, the control group, the blank group and the experimental group, respectively, and 800 is a multiple of dilution.

#### 2.5.3. The Inhibitory Effect of AAG-3 During In Vitro Digestion on α-Glucosidase

The inhibitory activity of α-glucosidase was determined according to the method of Jia with some modifications [25]. The samples, enzymes and substrates used in the reaction were dissolved in 0.1 mol/L PBS (pH 6.8). Following this, 100 μL of α-glucosidase solution (1 U/mL) and 100 μL of AAG-3/AAG-3-S/AAG-3-G/AAG-3-I (3 mg/mL) or different concentrations of AAG-3-I (0.2, 0.6, 1.0, 1.4, 1.6, 1.8 mg/mL) were incubated at 37 °C for 10 min. Then, 100 μL of 5 mM, *p*-nitrophenyl-α-*D*-glucopyranoside (PNPG) solution was added and incubated at 37 °C for 1 h. Finally, 1 mL of 0.2 M Na_2_CO_3_ was added to terminate the reaction. The blank group, control group and blank control group refer to the no α-glucosidase sample, no glycoprotein sample and no α-glucosidase and glycoprotein sample in the system, respectively. The inhibition rate of α-glucosidase was calculated using the following equation:

Inhibition rate = [1 − (A_1_ − A_2_)/(A_3_ − A_4_)] × 100%

where A_1_, A_2_, A_3_ and A_4_ represent the absorbance values of the experimental group, blank group, control group and blank control group, respectively.

#### 2.5.4. Hypoglycemic Activity of AAG-3-I on Insulin-Resistant HepG2 Cell Models

##### Cell Culture

HepG2 cells were cultured in DMEM with phenol red and supplemented with 10% (*v*/*v*) FBS, 1% streptomycin and penicillin, at 37 °C in a humidified atmosphere containing 5% CO_2_. The medium was changed every 24 h.

##### Cell Viability

The effect of the AAG-3-I on HepG2 cell viability was measured using a conventional Cell Counting Kit-8 (Dojindo, Tokyo, Japan). HepG2 cells were seeded in 96-well plates (1 × 10^4^ cells/well) for 24 h and then treated with 100 μL of AAG-3-I at varying concentrations of 200, 500, 800 and 1000 μg/mL for another 24 h. Then, 10 μL of CCK-8 solution (5 mg/mL) was added to the cells and incubated for 4 h. Cell viability was determined by measuring the absorbance at 450 nm.

##### Establishment of Insulin Resistance Model

To determine the optimal dose of insulin, HepG2 cells were cultured in different concentrations of insulin (including 5 × 10^−5^, 5 × 10^−6^, 5 × 10^−7^, 5 × 10^−8^ and 5 × 10^−9^ M) for 24 h [2]. The cell supernatants were collected. The glucose consumption was measured using an assay kit to evaluate the effectiveness of insulin in promoting glucose uptake of HepG2 cells. Whichever concentration of insulin leads to the highest glucose consumption rate of HepG2 cells is the best concentration to induce insulin resistance of HepG2 cells.

##### Glycogen Content Analysis

The HepG2 cells were cultured as described in Section Cell Culture and then treated as described above in Section Cell Viability. The cells were collected in a centrifuge tube and the supernatant was discarded after centrifugation. The cells were ultrasonically broken with 0.75 mL of the extraction buffer and then transferred to 10 mL tubes and placed in a 95 °C water bath for 20 min and vibrated every 5 min. After cooling, it was diluted to 5 mL with distilled water and then centrifuged at 4000 rpm for 10 min. The cell supernatants were collected for glycogen content analysis using a commercial kit following the manufacturer’s instruction.

##### PAS Staining

The PAS stain used a PAS stain kit following the manufacturer’s instruction. The cells were placed and treated as described above in Section Glycogen Content Analysis. Firstly, the cells were fixed in PAS fixative for 15 min, washed and dried. Secondly, the cells were oxidized using an oxidant at room temperature for 20 min and then rinsed twice with tap water and ionized water, respectively. The slice was covered after adding the Schiff’s reagent and placed in a dark place at room temperature for 10–20 min. Finally, the slice was re-stained in hematoxylin staining solution for 1–2 min, rinsed with running water for 2 min, dried and observed under an optical microscope.

##### Glucose Consumption Analysis

The HepG2 cells were divided into 7 groups: normal control group (NC), insulin-resistant model group (IR), positive control group (PC) and AAG-3-I different dose groups. Except for the normal control group, the insulin resistance cell model was established in other groups with the optimal resistance concentration (1 × 10^−6^ M). The positive control group were treated with 100 μg/mL of dimethyldiguanide. The AAG-3-I dose groups were treated with different concentrations of AAG-3-I (including 100, 200, 400 and 600 μg/mL). All the cells were incubated at 37 °C for 24 h. The cell supernatants were collected for glucose consumption analysis using a commercial kit following the manufacturer’s instruction.

##### HK and PK Activity Analysis

The cells were placed and treated as described above in Section Glycogen Content Analysis. The cells were collected in a centrifuge tube, and the supernatant was discarded after centrifugation. The activity of HK and PK were determined using commercial kits following the manufacturer’s instruction.

### 2.6. Statistical Analysis

All experiments were conducted with a minimum of three replicates. The results were expressed as mean ± standard deviation. Statistical comparison of means among multiple groups was done using a one-way analysis of variance (ANOVA) and LSD test. Data were analyzed using SPSS software (version 25.0, SPSS Inc., Chicago, IL, USA). Values of *p* < 0.05 were considered statistically significant.

## 3. Results and Discussion

### 3.1. Purification of AAG

**Figure 1:**
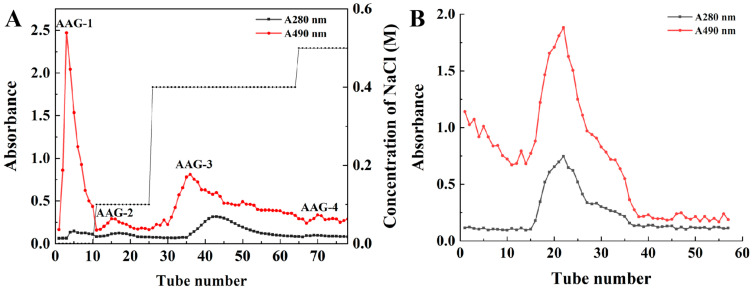
DEAE-Sepharose Fast Flow column chromatography of AAG (**A**); Sephadex G-75 column chromatography of AAG-3 (**B**).

The crude AAG was separated and purified using a DEAE-Sepharose Fast Flow column. An elution curve with 0–0.7 M NaCl Tris-HCl buffer (pH 8.0) showed that the four elution overlap peaks of protein and carbohydrates appeared at the NaCl concentration of 0 M, 0.1 M, 0.4 M and 0.5 M, indicating that four glycoprotein components (AAG-1, AAG-2, AAG-3 and AAG-4) were eluted (Figure 1A). The elution protein peaks of AAG-1, AAG-2 and AAG-4 were too small to be collected. Therefore, AAG-3 was collected and obtained after dialysis and freeze-drying.

AAG-3 was further purified using Sephadex G-75 and eluted with deionized water. One elution overlap peak of protein and carbohydrates appeared (Figure 1B), suggesting that AAG-3 was glycoprotein.

### 3.2. Structural Analysis of AAG-3

#### 3.2.1. Protein Content and Carbohydrate Content of AAG-3

**Figure 2:**
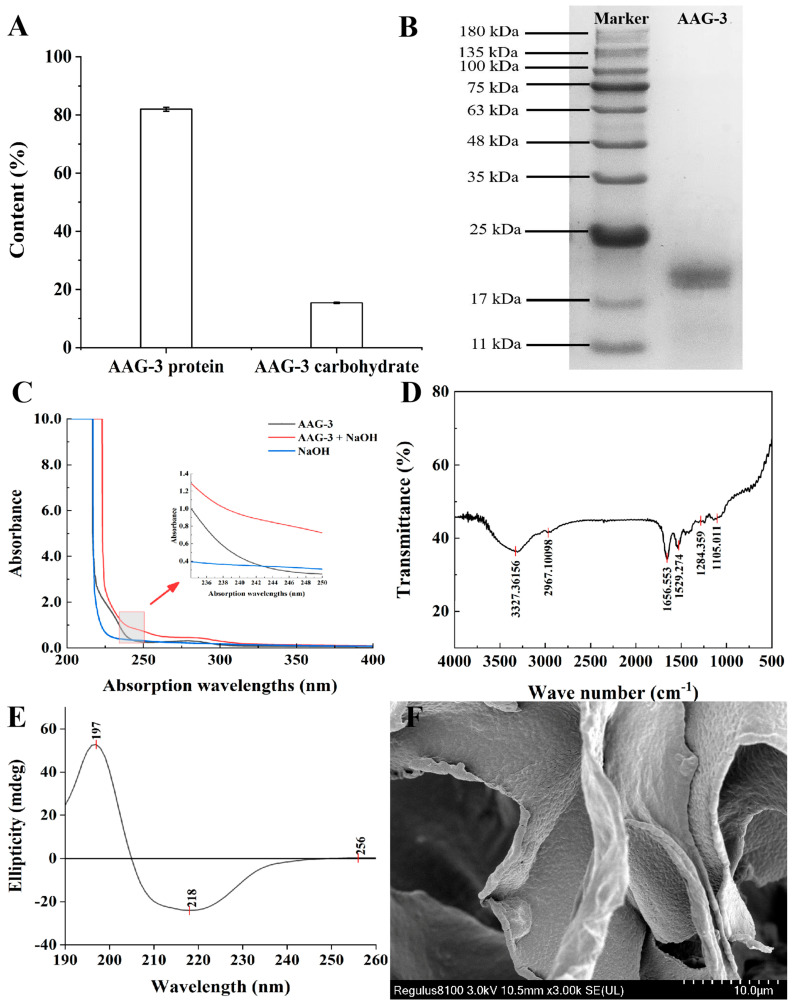
Structural characterization of AAG-3. Protein content and carbohydrate content of AAG-3 (**A**); The SDS-PAGE of AAG-3 (**B**); UV spectrum of AAG-3 with or without NaOH treatment (**C**); FTIR spectrum of AAG-3 (**D**); CD spectrum of AAG-3 (**E**); SEM image of AAG-3 (3000×) (**F**).

The protein content and carbohydrate content of AAG-3 were 81.96% and 15.40%, respectively (Figure 2A). The purity of AAG-3 was above 96%. However, the protein content and carbohydrate content of glycoprotein (lectin) extracted from *A. auricula* by Liu et al. were 20.9% and 10.3%, respectively, which were different from that of AAG-3 [14].

#### 3.2.2. SDS-PAGE of AAG-3

SDS-PAGE electrophoresis of AAG-3 showed that AAG-3 was a single glycoprotein component with short polysaccharide chains and its Mw was between 17 kDa and 20 kDa (Figure 2B). According to the relative mobility and the logarithm of the Mw of protein standards from 180 kDa to 10 kDa (Table 1), the linear regression equation was Y = 2.28254 − 1.35909X, and the correlation coefficient was R^2^ = 0.99163 > 0.99, which indicated that the results were credible. According to the linear equation, the Mw of AAG-3 was approximately 18.21 kDa. It was different from the lectin (25 kDa) extracted from *A. auricula* by Liu et al. [14].

#### 3.2.3. Amino Acid Composition of AAG-3

AAG-3 contained 18 different types of amino acid and its total amino acid content was 64.416 g/100 g (Table 2). AAG-3 mainly contains alanine, aspartic acid, threonine, lysine, glutamic acid and so on.

In addition, AAG-3 contained 8 essential amino acids, and the content reached 26.657 g/100 g, accounting for 41.38% of the total amino acids. It was higher than the ideal protein standard required by the FAO/WHO, indicating that AAG-3 is a good source of essential amino acids for the human body. Threonine and serine, which constitute the O-glycopeptide bond of glycoprotein, accounted for 15.38% of the total amino acid content. The content of aspartic acid was 7.157 g/100 g. Aspartic acid can regulate the metabolic function of the brain and nerves, and its levorotatory is widely used in antidote, liver function promoter, fatigue recovery agent and other medical supplies [26].

#### 3.2.4. UV Spectrum Analysis of AAG-3

There are two main types of glycopeptide bonds in glycoproteins: N-type glycopeptide bonds and O-type glycopeptide bonds. The N-type glycopeptide bond is stable to alkaline solution, and the O-type glycopeptide bond is easily hydrolyzed by alkaline solution. After the glycoprotein is hydrolyzed in alkaline solution, the monosaccharide or polysaccharide chain linked to the serine or threonine hydroxyl group on the peptide chain are hydrolyzed, resulting in the O-glycopeptide bond undergoing a β-elimination reaction. The corresponding serine and threonine are converted into α-aminoalanine and α-aminobutenoic acid, resulting in enhanced UV absorption at 240 nm. In Figure 2C, the NaOH solution alone did not cause an enhanced absorption peak at 240 nm. Compared with the aqueous solution of AAG-3, the AAG-3 treated with dilute NaOH had a significant absorption enhancement at 240 nm, indicating that the β-elimination reaction occurred and there was an O-type glycopeptide bond in AAG-3 [8].

#### 3.2.5. FTIR Spectrum Analysis of AAG-3

The infrared spectrum of AAG-3 is shown in Figure 2D. The absorption peak between 3500 cm^−1^ and 3200 cm^−1^ was the stretching vibration of O-H and N-H, indicating the existence of intermolecular and intramolecular hydrogen bonds [3]. The peak at 2967 cm^−1^ was attributed to the stretching vibration of C-H bond in the polysaccharide. The strong absorption peaks around 1656 cm^−1^ and 1529 cm^−1^ were attributed to the C=O bond stretching vibration and the N-H bond bending vibration on the amide group (-CONH_2_) [3,14,27], which were the characteristic absorption peaks of amino polysaccharide. The strong absorption peak near 1284 cm^−1^ was the stretching vibration of the C-N bond on the amine [14]. The peak at 1105 cm^−1^ was attributed to the vibrational absorption of C-O bond in the polysaccharide [3].

#### 3.2.6. CD Spectrum Analysis of AAG-3

The secondary structure type and content of glycoprotein can be investigated by measuring the CD spectrum of glycoprotein in the far ultraviolet region (190–250 nm) [22]. Different types of secondary structure have different absorption peak characteristics in this region: α-helix has a positive peak near 190 nm and a negative peak near 208 nm and 222 nm; β-sheet has a positive peak near 195 nm and a negative peak near 218 nm [28]. The random coil had a negative peak near 198 nm and a small and broad positive peak near 212 nm [8]. AAG-3 had a positive peak near 200 nm and a negative peak at 208–222 nm but no positive peak near 220 nm, indicating the presence of α-helix and β-sheet but a lack of a random coil in AAG-3 (Figure 2E).

The content of each secondary structure in AAG-3 was further analyzed using software. The α-helix and β-sheet with high stability are ordered structures, while the β-turn and random coil are disordered structures [29]. The results showed that the secondary structures of AAG-3 were mainly composed of many α-helixes and β-turns and few β-sheets, suggesting that AAG-3 had strong structural stability and few β-sheet lamellar structures (Table 3). High structural stability may enable glycoproteins to freely pass through biofilms and ensure their active conformation, thus enhancing the hypoglycemic activity by increasing the bioavailability [29]. These results suggest that AAG-3 has good hypoglycemic activity.

#### 3.2.7. SEM Analysis of AAG-3

SEM analysis showed that AAG-3 had a lamellar structure with spiral folds, which might be related to AAG-3 containing many α-helix and β-turn secondary structures (Figure 2F).

### 3.3. In Vitro Hypoglycemic Activity

#### 3.3.1. The Inhibitory Effect of AAG-3 During In Vitro Digestion on α-Amylase

**Figure 3:**
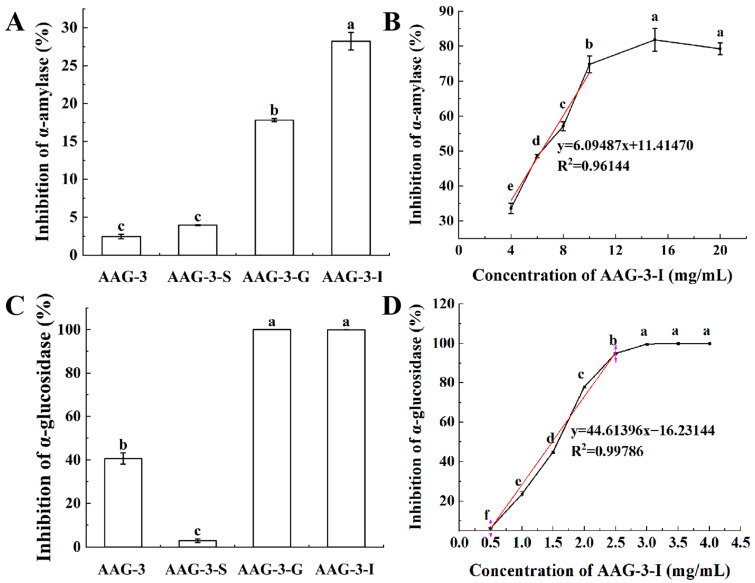
The inhibitory effects of AAG-3 during in vitro digestion on α-amylase (**A**) and α-glucosidase (**C**); the inhibitory effects of different concentrations of AAG-3-I on the α-amylase (**B**) and α-glucosidase (**D**). Data are presented as mean ± SD. Different lowercase letters indicate significant differences (*p* < 0.05) in multifactorial analyses among the groups.

The α-amylase secreted by the salivary glands or pancreas releases glucose from starch into the blood. In this study, the effects of AAG-3 on the inhibition rate of α-amylase after the simulated salivary, gastric and intestinal digestion environment were explored (Figure 3A). The results showed that AAG-3 itself had a low inhibitory effect on α-amylase activity. The inhibitory effect of AAG-3 on α-amylase may be due to its lower Mw. Lower Mw of glycoprotein can expose more active sites to enhance the binding ability of the enzyme, resulting in a change of the enzyme conformation to the loss of some activity of the enzyme [8].

After the simulated salivary digestion, the inhibition rates of AAG-3-S on α-amylase activity were higher than that of AAG-3, but there was no significant difference. After simulated gastric and intestinal digestions, the inhibition rates of AAG-3-G and AAG-3-I on α-amylase were significantly higher than that of AAG-3. Pepsin specifically targets hydrophobic amino acids such as Phe, Trp amd Tyr, while the trypsin sites are Arg and Lys. The increased inhibitory activity of AAG-3-G and AAG-3-I on the α-amylase might be related to the release of these hydrophobic amino acids, peptides and polysaccharide chains after pepsin and trypsin hydrolysis of glycoprotein [3]. The inhibitory effects of AAG-3 on α-amylase during the simulated gastrointestinal digestion in vitro were always stronger than those of pea glycoprotein (PGP2) [3]. In addition, AAG-3 gradually increased the inhibition of α-amylase during the simulated gastrointestinal digestion in vitro, while pea glycoprotein (PGP2) showed the opposite trend [3]. These results suggest that AAG-3 is more beneficial than pea glycoprotein (PGP2) in the release of polysaccharide chains or glycopeptides that inhibit the activity of α-amylase during the simulated gastrointestinal digestion in vitro and thus has better hypoglycemic activity.

To further investigate the dose–effect relationship of AAG-3-I on the inhibition of α-amylase activity, the inhibitory rates of AAG-3-I on α-amylase activity were investigated at different concentrations (Figure 3B). When the concentration of AAG-3-I was in the range of 4–15 mg/mL, the inhibition rate of AAG-3-I on α-amylase significantly increased from 33.59% to 81.79%. The median inhibitory concentration (IC50) of AAG-3-I for the inhibition of α-amylase activity was 6.33 mg/mL.

#### 3.3.2. The Inhibitory Effect of AAG-3 During In Vitro Digestion on α-Glucosidase

The α-glucosidase secreted in the small intestine can catalyze the hydrolysis of the non-reducing α-(1-4) glycosidic bond to release glucose from maltose or sucrose into blood resulting in post-prandial hyperglycemia [3]. Therefore, inhibition of α-glucosidase activity is a potential effective strategy to control postprandial blood glucose in the treatment and prevention of T2DM. In this study, the effects of AAG-3 on the inhibition rate of α-glucosidase after the simulated salivary, gastric and intestinal digestion environment were explored. The results showed that AAG-3 itself had a good inhibitory effect on α-glucosidase (Figure 3C). After simulated salivary digestion, the inhibitory rate of AAG-3-S on α-glucosidase was significantly reduced compared with AAG-3. The inhibitory effect of AAG-3 on α-glucosidase may be due to its O-glycopeptide bond, hydrophobic amino acid and aromatic amino acid contents. It was found that O-glycopeptide bond-linked glycoproteins have a strong inhibitory effect on α-glucosidase [8]. The content of aromatic amino acid and hydrophobic amino acid in glycoproteins was positively correlated with the inhibition of α-amylase and α-glucosidase [8]. The hydrophobic amino acid content of AAG-3 was 26.42%, and the aromatic amino acid content was 8.65%, suggesting that AGG-3 had a good inhibitory effect on α-amylase.

After simulated gastric and intestinal digestions, the inhibition rates of AAG-3-G and AAG-3-I on α-glucosidase were significantly higher than that of AAG-3, and their inhibition rates on α-glucosidase were close to 100%. This might be due to the digestion of pepsin and pancreatin leading to the polysaccharide chains and hypoglycemic peptides of the glycoprotein being released. Studies have shown that some polysaccharides have a good inhibitory effect on α-glucosidase [3,8]. In addition, studies have found that many active peptides also have good hypoglycemic activity [2,30]. An amount of 2 mg/mL AAG-3 could inhibit the activity of α-glucosidase by 77.92% after the simulated gastrointestinal digestion in vitro (Figure 3D). However, 2 mg/mL pea glycoprotein (PGP2) only inhibited the activity of α-glucosidase by 54.87% after the simulated gastrointestinal digestion in vitro [3]. These results suggest that AAG-3 maintains its hypoglycemic activity better than pea glycoprotein (PGP2) during gastrointestinal digestion.

To further investigate the dose–effect relationship of AAG-3-I on the inhibition of α-glucosidase activity, the inhibitory rates of AAG-3-I on α-glucosidase activity were investigated at different concentrations (Figure 3D). AAG-3-I showed a positive dose-dependent correlation with α-glucosidase inhibition in the range of 0.5–3.0 mg/mL. The median inhibitory concentration (IC50) of AAG-3-I for the inhibition of α-glucosidase activity was 1.48 mg/mL.

#### 3.3.3. Hypoglycemic Activity of AAG-3-I on Insulin-Resistant HepG2 Cells

##### Cell Viability of AAG-3-I

**Figure 4:**
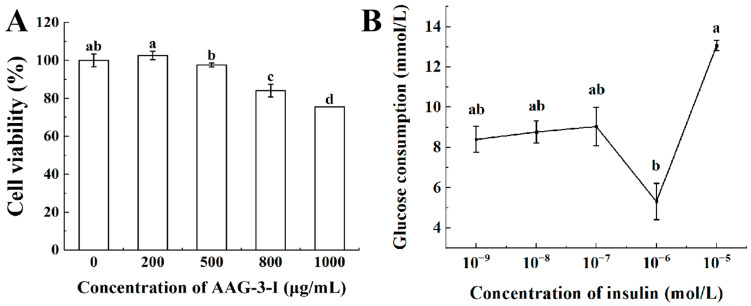
Effects of different concentrations of AAG-3-I on the viability of HepG2 cells (**A**); effects of different concentrations of insulin on the glucose consumption of HepG2 cells (**B**). Data are presented as mean ± SD. Different lowercase letters indicate significant differences (*p* < 0.05) in multifactorial analyses among the groups.

The effects of different concentrations of AAG-3-I on the viability of HepG2 cells were determined via the CCK method. In the range of 200–800 μg/mL, AAG-3-I concentration was negatively correlated with HepG2 cell activity (Figure 4A). Through linear regression equation analysis, the upper limit of AAG-3-I concentration required to maintain HepG2 cell activity above 90% was 621 μg/mL. Therefore, AAG-3-I within 600 μg/mL was selected for the following experiments. The tolerance of cells is different for different glycoproteins. The glycoproteins (PGP1 and PGP2) obtained from pea (*Pisum sativum* L.) showed strong cytotoxicity of cells at 250 μg/mL. Therefore, the cells have a stronger tolerance to AAG-3-I, suggesting that AAG-3-I can be applied to a larger dose of hypoglycemic functional food to have a better hypoglycemic effect.

##### Establishment of Insulin Resistance Model

To model insulin resistance, the effects of different concentrations of insulin on glucose consumption were investigated. When the concentration of insulin increased to 10^−6^ M, the glucose consumption of HepG2 cells decreased significantly (*p* < 0.05), indicating that the cells had insulin resistance (Figure 4B). When the insulin concentration was further increased to more than 10^−5^ M, the glucose consumption of HepG2 cells increased again (*p* < 0.05), suggesting that the cells might have undergone adaptive changes and become sensitive to high insulin concentration again. Therefore, the optimal insulin concentration for establishing the insulin resistance model of HepG2 cells was 1 × 10^−6^ M.

##### Glycogen Content and PAS Staining Analysis of AAG-3-I

**Figure 5:**
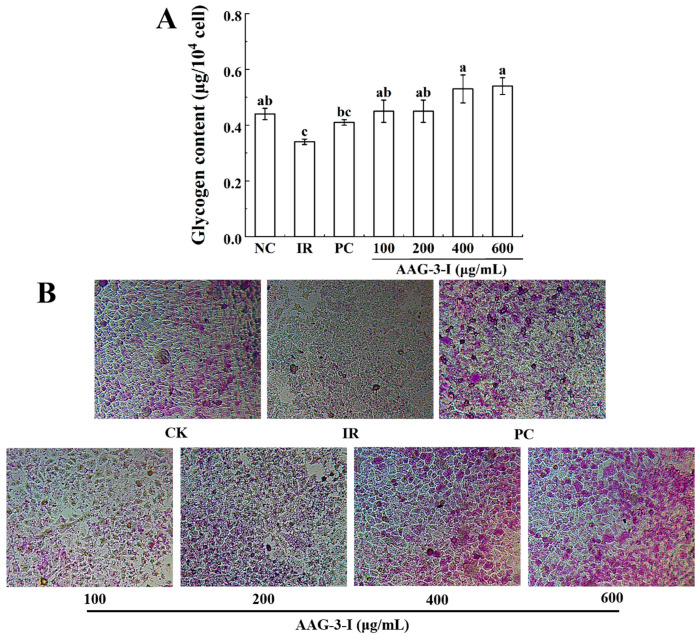
Effects of different concentrations of AAG-3-I on the glycogen content of insulin-resistant HepG2 cells (**A**). Data are presented as mean ± SD. Different lowercase letters indicate significant differences (*p* < 0.05) in multifactorial analyses among the groups. Effects of different concentrations of AAG-3-I on the glycogen PAS staining of insulin-resistant HepG2 cells (**B**).

The results showed that the glycogen content in the IR model group was significantly lower than that in the NC group (*p* < 0.05) (Figure 5A) Hepatic insulin resistance is usually characterized by reduced cellular glycogen synthesis [2]. AAG-3-I could significantly promote glycogen synthesis at different concentrations (*p* < 0.05), and the effect of AAG-3-I on promoting glycogen synthesis reaches the maximum when its concentration reached 600 μg/mL.

The effect of AAG-3-I on glycogen synthesis in insulin-resistant HepG2 cells was further observed via glycogen PAS staining. PAS can stain glycogen purplish red. AAG-3-I can promote glycogen synthesis in insulin-resistant HepG2 cells, in a dose-dependent manner (Figure 5B). These results suggested that AAG-3-I could effectively promote the recovery of glycogen synthesis in insulin resistant cells.

##### Glucose Consumption Analysis of AAG-3-I

**Figure 6:**
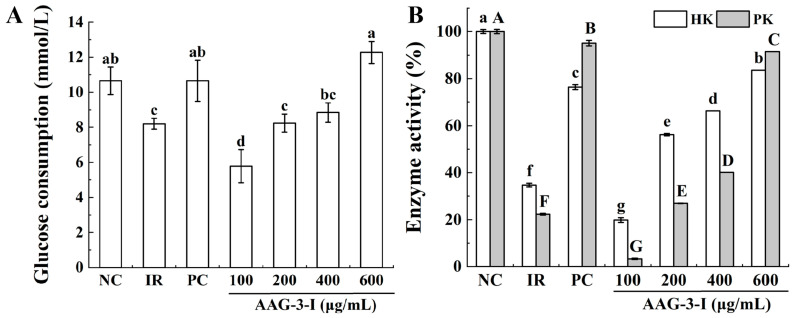
Effects of different concentrations of AAG-3-I on the glucose consumption of insulin-resistant HepG2 cells (**A**); effects of different concentrations of AAG-3-I on the HK and PK activities of insulin-resistant HepG2 cells (**B**). Data are presented as mean ± SD. Different letters indicate significant differences (*p* < 0.05) in multifactorial analyses among the groups.

The results showed that glucose consumption in the IR model group was significantly lower than that in the NC group (*p* < 0.05) (Figure 6A), suggesting that the IR cell model was established successfully. Glucose consumption in the insulin-resistant cell was significantly increased via the treatment of AAG-3-I at the concentration of 600 μg/mL, indicating that AAG-3-I might effectively improve glucose metabolism of insulin-resistant cells.

##### HK and PK Enzyme Activities Analysis of AAG-3-I

HK and PK are important rate-limiting enzymes of glycolysis in the liver and play a key role in glucose metabolism [2]. The results showed that HK and PK activities in the IR model group were significantly decreased as compared to the NC group (*p* < 0.05) (Figure 6B). When the concentration of AAG-3-I was greater than 200 μg/mL, it could promote the glycogen synthesis of insulin-resistant HepG2 cells in a dose-dependent effect. These results suggested that AAG-3-I could enhance the activities of HK and PK in insulin-resistant HepG2 cells, thereby increasing the rate of glycolysis, decreasing the level of glucose in blood and improving insulin resistance.

##### Potential Mechanism of AAG-3-I to Improve Insulin-Resistant HepG2 Cells

Insufficient expression or abnormal phosphorylation of IRS2 play an important role in the occurrence of IR and T2DM [29]. Akt can regulate the insulin signaling and glucose metabolism through promoting glycogen synthesis, affecting key enzymes (HK and PK) activities in glycolysis/gluconeogenesis, transporting Glut2 to the outer membrane of hepatocytes to improve its utilization and improving the glycogen storage [29]. Glycoproteins could activate the IRS2/Akt pathway in insulin resistance signaling, thereby regulating glucose metabolism [29]. In our study, AAG-3-I could improve cellular glycogen synthesis and reduce gluconeogenesis in insulin-resistant HepG2 cells by increasing the glucose consumption, glycogen content and HK and PK enzyme activities. Therefore, we speculated that AAG-3-I might improve glucose metabolism of insulin-resistant HepG2 cells through activation of IRS2/Akt signaling pathway, and this inference needs to be verified in future studies.

## 4. Conclusions

A glycoprotein AAG-3 with an Mw of 18.21 kDa was isolated from *Auricularia Auricula*, which had an O-type glycopeptide bond, 18 kinds of amino acid, many α-helixes and β-turns and few β-sheets and sheet-like microstructures. With the simulated digestion of AAG-3 in vitro, the inhibition of α-amylase and α-glucosidase activities as well as the glucose consumption, glycogen content and HK and PK enzyme activities in insulin-resistant HepG2 cells were significantly increased. Therefore, AAG-3 has a potential role in hypoglycemic activity by improving cellular glycogen synthesis and reduced gluconeogenesis in insulin-resistant cells. Its hypoglycemic activity may be linked to its low molecular weight, O-type glycopeptide, high structural stability (protein secondary structure α-helix and β-sheet content), aliphatic amino acid content, aromatic amino acid content and hydrophobic amino acid content. Collectively, AAG-3 has good potential for hypoglycemic activity and can be used as a potential nutritional supplement for diabetic patients. In the future, the hypoglycemic signaling pathways activated by AAG-3, the metabolic profile of AAG-3 in experimental animals and clinical trials need to be further investigated in order to expand the application of AAG-3 in functional hypoglycemic foods.

## Figures and Tables

**Table 1:** Protein Mw and relative mobility determination.

Gelation Length (Pixel): 16.2			
Standard Protein Mw (kDa)	Migration Distance (Pixels)	Relative Mobility (Rf)	Lg (MW)
180	6.6	1.4	0.086419753
135	5.9	2.1	0.12962963
100	5	3	0.185185185
75	4.4	3.6	0.222222222
63	3.4	4.6	0.283950617
48	1.8	6.2	0.382716049
35	0.3	7.7	0.475308642
25	−2.2	10.2	0.62962963
17	−5	13	0.802469136
10	−7	15	0.925925926
AAG-3	11.8	0.728395062	1.260341975
Relative mobility (Rf) = 2.28254 − 1.35909 Lg (MW), R^2^ = 0.99163			

**Table 2:** Amino acid composition and proportion of AAG-3.

Serial Number	Amino Acid Name	Abbreviation	Concentration Ratios (%)
1	Aspartic acid	Asp	7.16 ± 0.01
2	Threonine *	Thr	6.60 ± 0.01
3	Serine	Ser	3.31 ± 0.01
4	Glutamic acid	Glu	5.16 ± 0.01
5	Glycine #	Gly	2.30 ± 0.01
6	Alanine #	Ala	7.95 ± 0.01
7	Cysteine	Cys	1.09 ± 0.01
8	Valine *#	Val	2.91 ± 0.02
9	Methionine *#	Met	0.09 ± 0.01
10	Isoleucine *#	Ile	1.87 ± 0.01
11	Leucine *#	Leu	4.11 ± 0.01
12	Tyrosine $	Tyr	3.34 ± 0.02
13	Phenylalanine *#$	Phe	4.30 ± 0.02
14	Lysine *	Lys	5.77 ± 0.01
15	Histidine	His	2.79 ± 0.01
16	Arginine	Arg	1.77 ± 0.01
17	Proline #	Pro	2.89 ± 0.01
18	Tryptophan *$	Trp	1.01 ± 0.01
Total free amino acids		64.42 ± 0.18	
Essential amino acids *		26.66 ± 0.08	
Hydrophobic amino acids #		26.42 ± 0.10	
Aromatic amino acids $		8.65 ± 0.05	

* Essential amino acids; # Hydrophobic amino acids; $ Aromatic amino acids.

**Table 3:** Secondary structure content of AAG-3.

Secondary Structure	α-Helix	β-Sheet	β-Turn	Random Coil
Content (%)	57.55 ± 0.65	11.00 ± 0.70	31.45 ± 0.05	0

## Data Availability

The original contributions presented in the study are included in the article, further inquiries can be directed to the corresponding author.
